# A study of Epstein-Barr virus infection in the Chinese tree shrew(*Tupaia belangeri chinensis*)

**DOI:** 10.1186/s12985-017-0859-5

**Published:** 2017-10-06

**Authors:** Zhi Wang, Xiang Yi, Long Du, Hong Wang, Jie Tang, Menglin Wang, Chenglin Qi, Heng Li, Yongjing Lai, Wei Xia, Anzhou Tang

**Affiliations:** 1 Department of Otorhinolaryngology Head and Neck Surgery, The First Affiliated Hospital of Guangxi Medical University, Nanning, Guangxi China; 2 Key Laboratory of Early Prevention and Treatment for Regional High Frequency Tumor, Ministry of Education, Nanning, Guangxi China

**Keywords:** Epstein–Barr virus, Tree shrews, Animal model, Infection

## Abstract

**Background:**

Epstein–Barr virus (EBV) is closely associated with many human diseases, including a variety of deadly human malignant tumours. However, due to the lack of ideal animal models,the biological characteristics of EBV, particularly its function in tumourigenesis, have not been determined. Chinese tree shrews (*Tupaia belangeri chinensis*), which are similar to primates, have been used to establish a variety of animal models and have recently received much attention. Here, we established tree shrews as a model for EBV infection by intravenous injection.

**Methods:**

Ten tree shrews were inoculated with EBV by intravenous injection,and blood was collected at regular intervals thereafter from the femoral artery or vein to detect EBV markers.

**Results:**

Eight of 10 tree shrews showed evidence of EBV infection. In the 8 EBV-infected tree shrews, EBV copy number increased intermittently or transiently, EBV-related gene expression was detected, and anti-EBV antibodies increased to varying degrees. Macroscopic hepatomegaly was observed in 1 tree shrew, splenomegaly was observed in 4 tree shrews, and enlarged mesenteric lymph nodes were observed in 3 tree shrews. Haematoxylin and eosin (HE) staining showed splenic corpuscle hyperplasia in the spleens of 4 tree shrews and inflammatory cell infiltration of the liver of 1 tree shrew and of the mesenteric lymph nodes of 3 tree shrews. EBER in situ hybridization(ISH) and immunohistochemical (IHC) staining showed that EBER-, LMP1- and EBNA2- positive cells were present in the spleens and mesenteric lymph nodes of some tree shrews. Western blotting (WB) revealed EBNA1-positive cells in the spleens of 4 tree shrews. EBV markers were not detected by HE, EBER-ISH or IHC in the lung or nasopharynx.

**Conclusions:**

These findings suggest that EBV can infect tree shrews via intravenous injection. The presented model offers some advantages for exploring the pathophysiology of EBV infection in humans.

## Background

Epstein-Barr virus (EBV) was first isolated from Burkitt lymphoma cells in 1964. The virus targets B lymphocytes and belongs to the human herpesvirus 4 family. The virus is wide spread,and more than 90% of adults are infected and are carriers for life [1]. EBV is spread through saliva and can also be transmitted through blood or sexual contact [2]. Its growth cycle includes two forms: a lytic period and a latent period. The lytic period, which is the proliferation period of EBV, consists of three consecutive phases with immediate early (IE)-, early (E)- and late (L)-expressing genes. The IE genes includeBZLF1(Zta)and BRLF1(Rta),the first transcription factors to appear during the lytic stage switch. The viral DNA is completely replicated for assembly during this period [3]. The latent period is the non-proliferative stage of EBV and is divided into three states according to the different latent genes that are expressed. Primary EBV infection passes through all three latent states before final co-existence with the host for long-term stability. When EBV infects resting-state B cells, it often establishes latency III and expresses all latent genes, including 3 latent membrane protein genes (LMPs), 6 nuclear antigen genes, and 2 non-protein-encoding RNAs (EBERs, EBER-1and EBER-2) [4]. The infected B cells then differentiate into memory B cells and proliferate. When the memory B cells that carry EBV enter the germinal centre B cells (centroblasts and centrocytes), latency II emerges with limited latent gene expression (LMPs, EBNA1, BARTs and EBERs). Latency I, which involves EBNA1 and EBERs expression,has been detected in proliferating memory B cells, and latency 0, without EBV protein expression but with the presence of non-translated viral RNAs, was demonstrated in resting memory B cells [5]. In vitro, EBV can specifically transform human peripheral blood B cells to EBV-infected latency III immortalized cell lines (lymphoblastoid cell lines, LCLs [6], and latent EBV proteins directly or indirectly play a key role in promoting the proliferation and transformation of the infected cells during this process [7–11].

Compared with infection in vitro, the mechanism of EBV infection in vivo is more complex and poorly understood. EBV is a tumourigenic virus, and the various latent infection states are also associated with different diseases, such as post-transplantation lymphoid cell hyperplastic diseases (post-transplantation lymphoproliferative disorders, PTLD) caused by the proliferation of infected cells and primarily due to EBV latency III infection, Hodgkin’s disease and nasopharyngeal carcinoma due to EBV latency II infection,and Burkitt lymphoma due to latency I infection. EBV is also associated with infectious mononucleosis, EBV-associated haemophagocytic syndrome and gastric carcinoma [12]. However, due to the lack of ideal animal models, the biological characteristics of EBV, particularly its function in tumourigenesis, have not been determined. Therefore, it is necessary to establish a suitable experimental EBV animal model.

Since the discovery of EBV, animal models of EBV infection have been established in various species, including primates, severe combined immune-deficiency (SCID) mice, transgenic animals, rabbits and guinea pigs. A variety of lymphomas have been induced using EBV continuous infection in cotton-top marmoset animal models [13–15]. B lymphocyte tissue hyperplasia and lymphoma have been observed in EBV animal models based on human peripheral blood lymphocytes (huPBLs)and the SCID mouse (hu-PBL-SCID mice) [16–19]. Studies of EBV using transgenic animal models have focused on individual genes such as EBNA1 and LMP1 [20–22]. Recent studies have confirmed that rabbits can be infected with EBV via venous and nasal inoculation [23–25] and that EBV can infect rabbit B cells in vitro [26]. Although these animal models have played important roles in studying the EBV infection process, its pathogenic mechanism, and its prevention and treatment, they also present some disadvantages. For human diseases, animal models of EBV infection involving non-human primates have irreplaceable advantages. Therefore, an EBV animal model that resembles human disease is urgently needed for the study of the biological characteristics of EBV and its associated diseases.

In this study, tree shrews (*Tupaia belangeri chinensis*) were used to establish an animal model of natural EBV infection. Tree shrews aresmall animals that are closely related to primates and live in tropical and subtropical regions; theirlife span is approximately eight years, and they are easily bred [27].Tree shrews are mainly distributed in Yunnan and Guangxi provincesin China and have been increasingly used in scientific and biomedical research in recent years. The Kunming Institute of Zoology recently analysed the entire genome, transcriptome [28] and proteome [29] of tree shrews. The results confirmed that tree shrews are closely genetically related to primates and that they have a high degree of similarity to primates and humans in physiological anatomy, neural development, and psychological stress responses. Many of the characteristics of viral infection in tree shrews are also similar to those of humans, and tree shrews can be infected by hepatitis A virus (HAV), hepatitis B virus (HBV),hepatitis C virus (HCV), rotavirus (HRV), adenovirus (ADV), and herpes virus (HSV) [30–35]. In addition, tree shrews have been used to establish animal models of drug-resistant bacterial infection and sepsis, psychological stress and depression, myopia, and metabolic disease [36–38]. Therefore, we studied whether tree shrews can be developed into a new type of animal model that is closer to primates than other existing animal models. Studies have shown that EBV hosts are limited to humans and New World monkeys, including the cotton-top marmoset, the common marmoset and the owl monkey [39] and that the infected cells are B lymphocytes and epithelial cells. Based on previous research and on the high similarity of tree shrews to primates, we proposed that tree shrews will be susceptible to Epstein-Barr virus infection and that they possess good potential for use as an animal model of EBV infection in humans.

## Results

### Effects of EBV inoculation in tree shrews

The effects of EBV inoculation in tree shrews are presented in Table 1. Thirteen tree shrews were used in this study, including 10 inoculated with 500 μl of EBV by intravenous injection and 3 inoculated with RPMI 1640 culture medium containing 10% foetal bovine serum using a similar procedure. Blood was collected prior to inoculation and every 1 or 2 weeks after inoculation. The number of copies of the EBV detected increased intermittently or transiently in the PBMCs of eight of the 10 tree shrews, and EBV-related antibodies increased by varying degrees. Animals Ts3 and Ts4 displayed severe weakness and were euthanized at week 12 and week 5. Another three of the 13 tree shrews (Ts1,2 and 8) died prior to the end of the experiment, but there was no evidence that the deaths resulted from EBV infection. The remaining 8 animals appeared to be normal and displayed no weakness after EBV inoculation. They were euthanized at times ranging from 3 to 21 weeks after inoculation with the virus, and their tissues were assayed for pathological changes.

**Table 1:** Summary of EBV infection in tree shrews

Tree shrew Number	EBV copies	EBV mRNA	EBV antibodies	EBERs	HE	IHC	WB	Observation period
Ts 1	+	+	+VCA IgG	–	–	–	–	4 weeks(dead) ^a^
Ts 2	+	+	+VCA IgG	+	+	–	+	6 weeks(dead) ^a^
Ts 3	+	+	+VCA IgG	+	+	–	+	12 weeks(euthanasia)
Ts 4	+	+	+VCA IgG	–	–	–	–	5 weeks(euthanasia)
Ts 5	–	–	–	–	–	–	–	21 weeks(euthanasia)
Ts 6	–	–	–	–	–	–	–	3 weeks(euthanasia)
Ts 7	+	+	+(VCA IgG、EBNA IgG)	+	+	+	–	21 weeks(euthanasia)
Ts 8	+	+	+VCA IgG	–	–	–	–	6 weeks(dead) ^a^
Ts 9	+	+	+VCA IgG	–	–	–	+	21 weeks(euthanasia)
Ts 10	+	+	+VCA IgG	+	+	+	+	3 weeks(euthanasia)
Negative control								
Ts 11	–	–	–	–	–	–	–	13 weeks(euthanasia)
Ts 12	–	–	–	–	–	–	–	5 weeks(euthanasia)
Ts 13	–	–	–	–	–	–	–	5 weeks(euthanasia)

+, positive; −, negative

^a^The 3 tree shrews that died prior to the end of the experiment may have died from infectious disease but the cause of death was undetermined

### EBV copy number in PBMCs of tree shrews post-infection

**Fig. 1:**
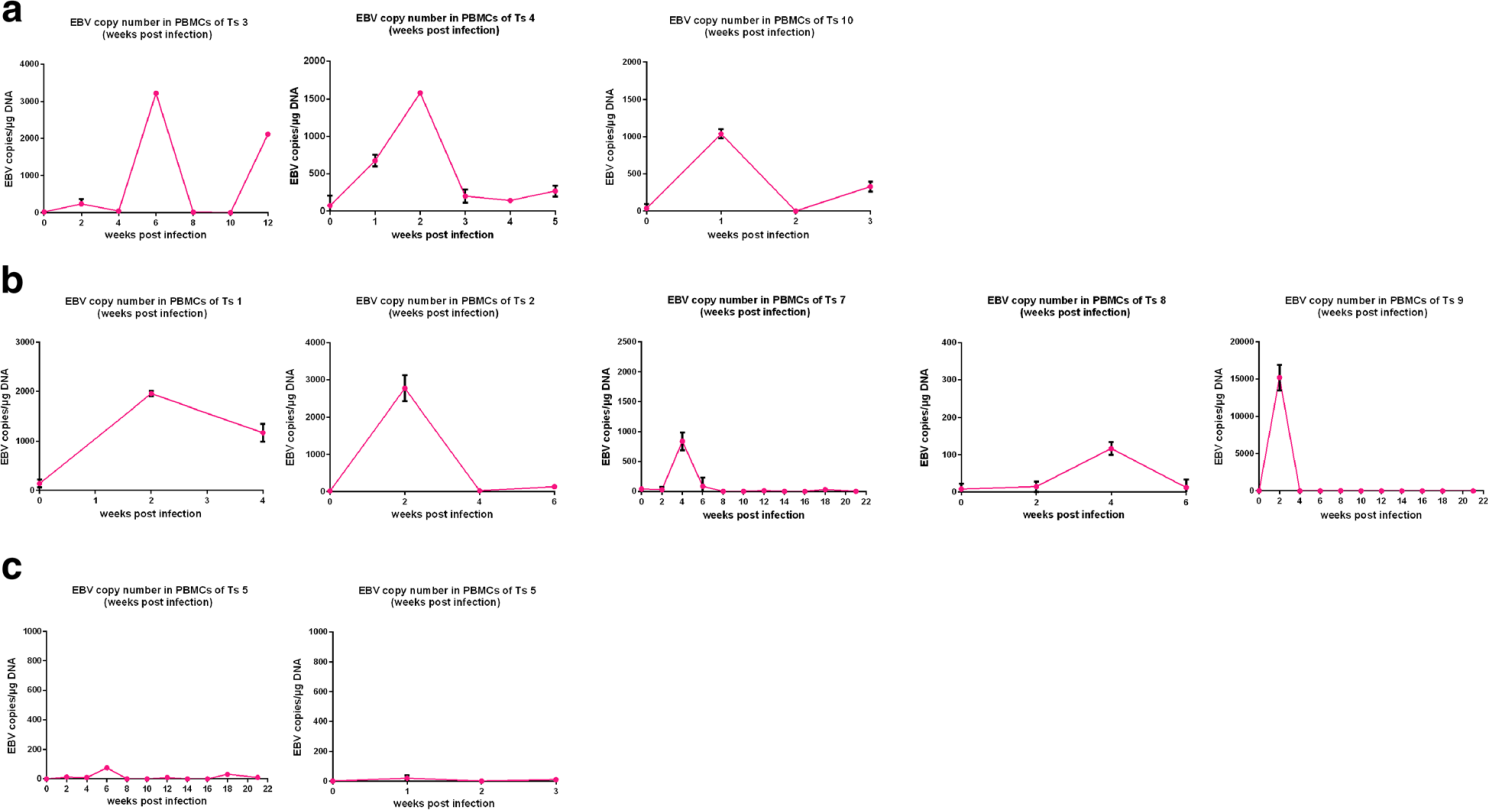
EBV copy number in PBMCs of tree shrews post-infection. In the 8 EBV-infected tree shrews, EBV copy number increased intermittently or transiently (**a**, **b**).The data for Ts5 and Ts6 are shown as representative of EBV-infection-negative tree shrews (**c**). The EBV copy number did not increase during the observation period in Ts5, Ts6 or Ts11-Ts13 (data not shown)

The EBV copy number in PBMCs (copies/μg DNA) was determined using qPCR. The results are shown in Fig. 1. Eight of the 10 tree shrews showed evidence of EBV infection. In 3 of the tree shrews, EBV copy number increased intermittently and appeared to fluctuate (Fig. 1a). However, in the other 5 tree shrews, EBV copy number increased to the highest value at week 2 or 4 and followed by a decline, as shown in Fig. 1b. Among the 8 infected tree shrews, the copy number of EBV was highest in Ts9 (12,233 copies/μg DNA); this represented a transient increase in the EBV copy number. The data for Ts5 and Ts6 are shown as representative of EBV-infected-negative tree shrews (Fig. 1c). In Ts5, Ts6 andTs11-Ts13, the EBV copy number did not increase during the observation period (data not shown).

### Expression of EBV genes in PBMCs of tree shrews post-infection

**Fig. 2:**
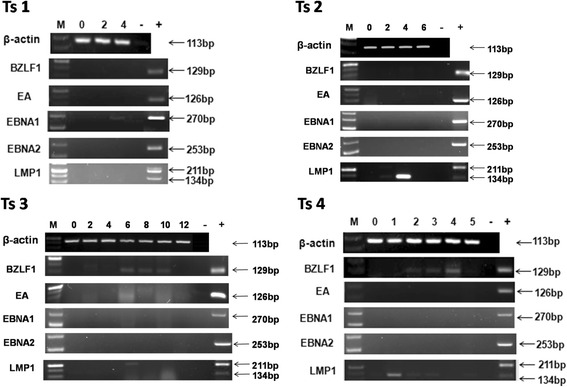
Expression of EBV genes in PBMCs of Ts1–4. In Ts1 and Ts2, only EBNA1 and LMP1 were detected at week 4, respectively. In Ts3, BZLF1 was expressed from weeks 6 to 10, and LMP1 was observed only at week 6.In Ts4, BZLF1 was expressed from weeks 2 to 4, and the expression of LMP1 persisted throughout the observation period with the exception of week 4

**Fig. 3:**
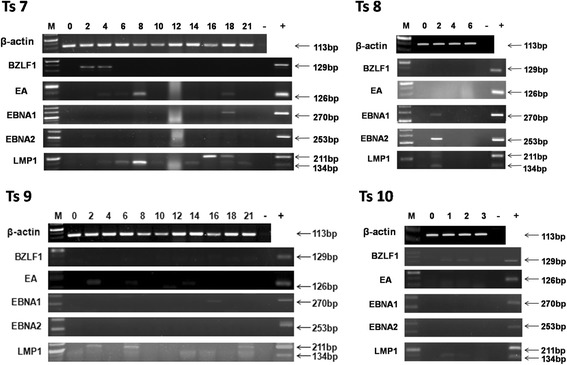
Expression of EBV genes in PBMCs of Ts7–10. In Ts7, the expression of LMP1 persisted throughout the observation period, excluding week 2, BZLF1 appeared at weeks 2 to 4,EA was detected intermittently from weeks 4 to 18, and EBNA1 was observed only at week 18. In Ts8, EBNA1, EBNA2 and LMP1were expressed at week 2. In Ts9, LMP1was detected at weeks 2, 6 and 21, and EA and EBNA1were detected only at weeks 2 and 16, respectively. In Ts10, the expression of BZLF1 persisted throughout the observation period,and LMP1 was detected at week 1

**Fig. 4:**
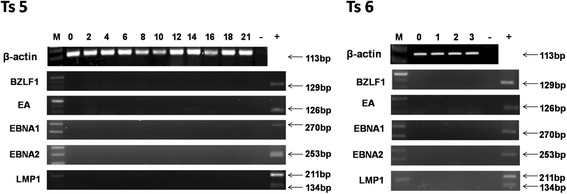
Expression of EBV genes in PBMCs of Ts5 and Ts6. No EBV gene expression was detected in Ts5 or Ts6 or in the 3 tree shrews that were used as negative controls (data not shown)

RT-PCR analysis of BZLF1, EA, EBNA1, EBNA2, and LMP1 was performed; the results are summarized in Figs. 2, 3 and 4.In Ts1 and Ts2, only EBNA1 and LMP1 were detected at week 4, respectively. In Ts3, BZLF1 was expressed from weeks 6 to 10, and LMP1 was observed only at week 6.In Ts4, BZLF1 was expressed from weeks 2 to 4, and the expression of LMP1 persisted throughout the observation period with the exception of week 4(Fig. 2). In Ts7, the expression of LMP1 persisted throughout the observation period, excluding week 2, BZLF1 appeared at weeks 2 to 4,EA was detected intermittently from weeks 4 to 18, and EBNA1 was observed only at week 18. In Ts8, EBNA1, EBNA2 and LMP1were expressed at week 2. In Ts9, LMP1was detected at weeks 2, 6 and 21, and EA and EBNA1were detected only at weeks 2 and 16, respectively. In Ts10, the expression of BZLF1 persisted throughout the observation period,and LMP1 was detected at week 1 (Fig. 3). None of these genes were detected in Ts5, Ts6 or in the3 tree shrews (data not shown) that were injected with RPMI 1640 culture medium containing 10%FBS as a negative control. β-actin was used as an internal control, and the cDNA of B95–8 cells was used as a positive control (Fig. 4).

### Levels of EBV antibody in the serum of tree shrews post-infection

**Fig. 5:**
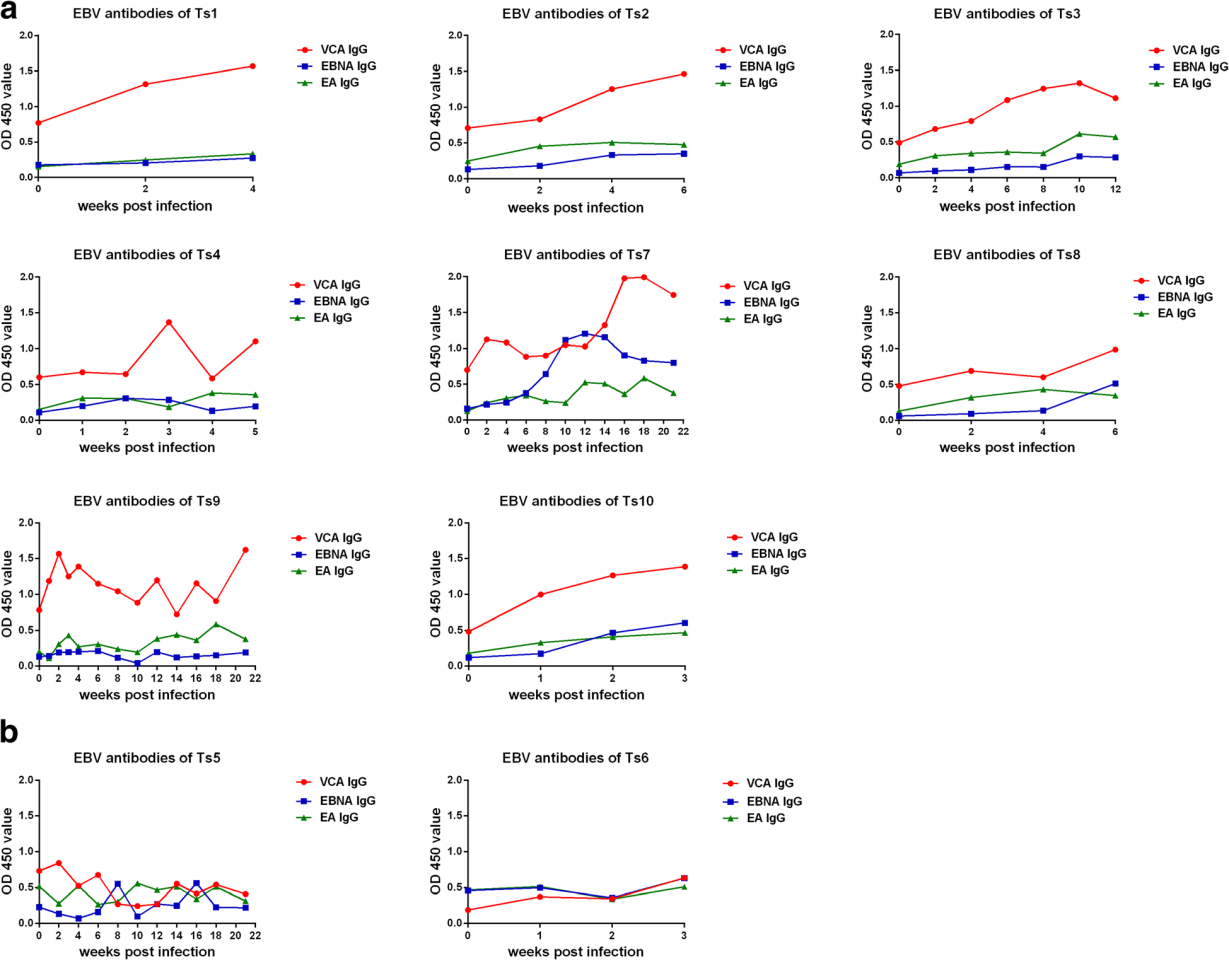
Levels of EBV antibody in the serum of tree shrews post-infection. The level of VCA IgG increased to varying degrees in all tree shrews in which the EBV copy number increased; EBNA1 IgG increased in only1 tree shrew (Ts7),and EA IgG was not elevated (**a**). The levels of these antibodies were not increased in the EBV-infection-negative tree shrews (**b**) or in any of the 3 tree shrews that served as negative controls (data not shown)

EBV antibodies in tree shrew serum were measured by ELISA. The results are shown in Fig. 5. The level of VCA IgG increased to varying degrees in all tree shrews in which the EBV copy number increased, but EBNA1 IgG increased only in 1 tree shrew (Ts7), and EA IgG was not elevated, as shown in Fig. 5a. The levels of these antibodies were not increased in the EBV-infection-negative tree shrews (Fig. 5b) or in any of the 3 tree shrews that served as negative controls (data not shown).

### Haematoxylin and eosin (HE) analysis of tree shrew tissues

**Fig. 6:**
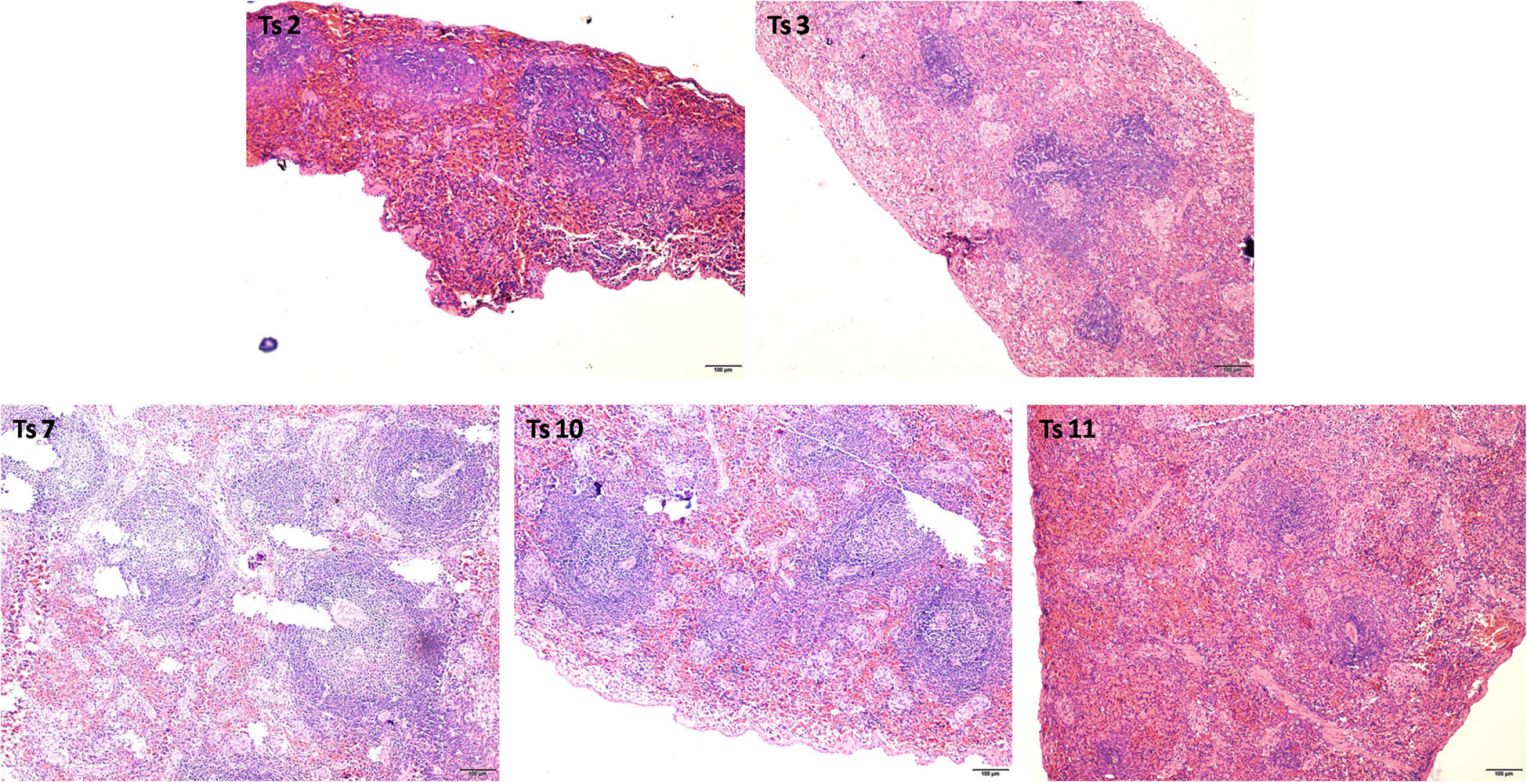
HE staining of the spleens of tree shrews. Splenic corpuscle hyperplasia was detected in the spleens of four (Ts2,3,7,10)of the 8 infected tree shrews and was most obvious inTs7.Ts11 is the negative control

**Fig. 7:**
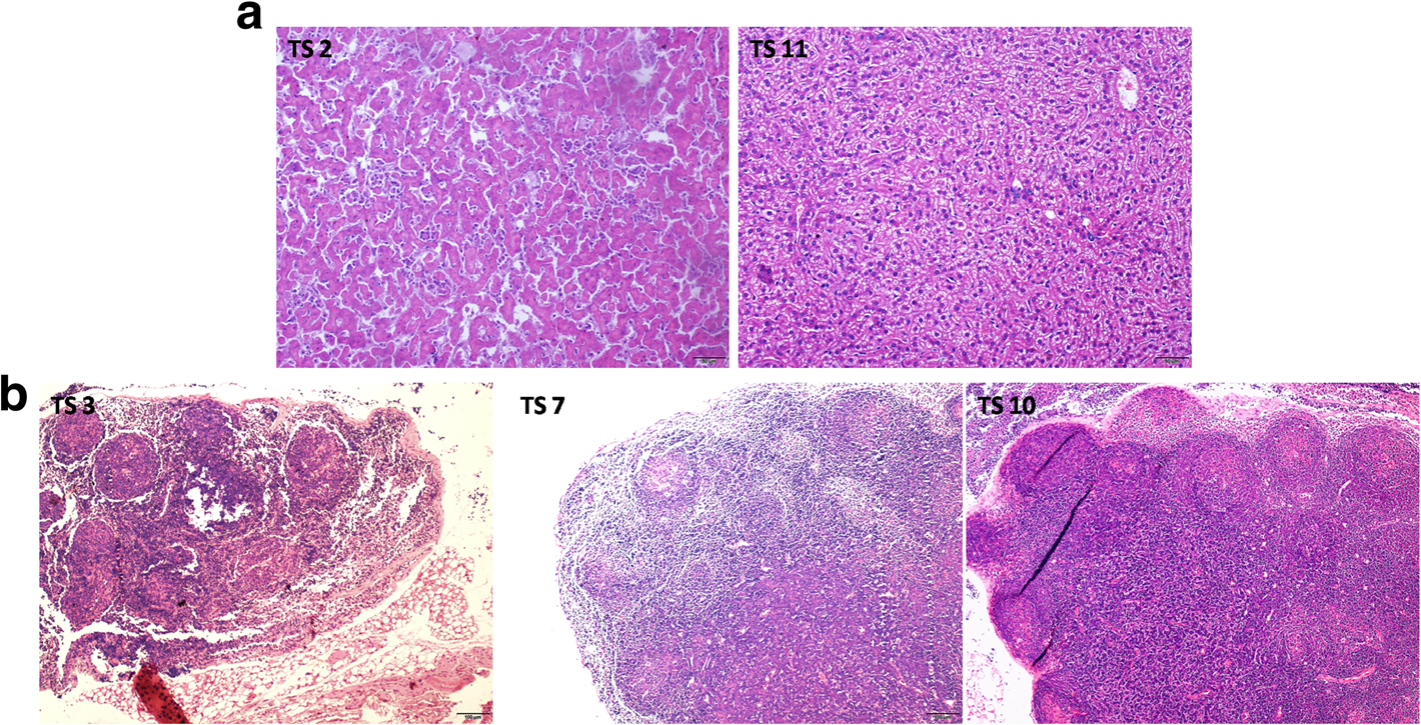
HE staining of the livers and mesenteric lymph nodes of tree shrews. Extensive inflammatory cell infiltration in the liver was detected in only 1(Ts2) of the 8 infected tree shrews (**a**). A large amount of lymphocyte proliferation was detected in 3 animals (Ts3,7 and 10) with mesenteric lymph node enlargement (**b**). Ts11 is the negative control

HE staining showed that compared with the negative controls, splenic corpuscle hyperplasia was detected in the spleens of four (Ts2,3,7 and 10)of the 8 infected tree shrews and was most obvious inTs7 (Fig. 6).In the liver, extensive inflammatory cell infiltration was detected in only 1(Ts2) of the 8 infected tree shrews (Fig. 7a).Extensive lymphocyte proliferation was observed in 3 animals (Ts3,7 and 10) with mesenteric lymph node enlargement (Fig. 7b). Ts11 served as the negative control. No obvious abnormalities in the lung or nasopharynx were detected after HE staining (data not shown).

### Detection of EBV in tree shrew tissues

**Fig. 8:**
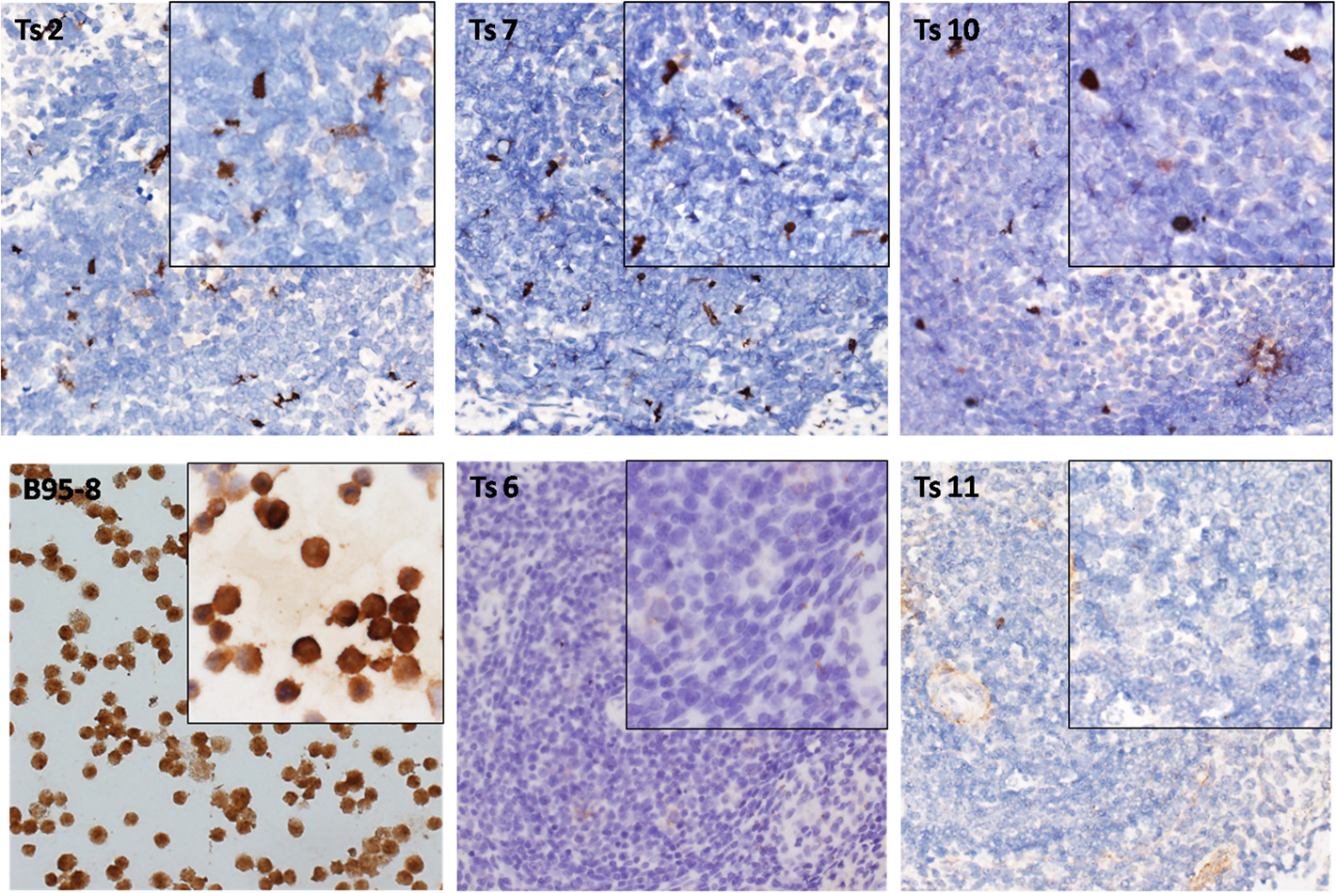
EBER-ISH staining of the spleens of tree shrews. EBER-positive cells were detected in the spleens of three of the ten tree shrews.B95–8 cells and Ts11 were used as positive and negative controls,respectively. Ts6 was an EBV-infection-negative tree shrew. No EBER-positive cells were detected inTs6 or Ts11

EBER-ISH was performed on 5-μm sections of formalin-fixed, paraffin-embedded tissue sections of the spleen, liver, mesenteric lymph nodes, lung and nasopharyngeal tissues of autopsied tree shrews. EBER-positive cells were detected in the spleen and mesenteric lymph nodes of 3 different tree shrews (Figs. 8 and 10a).EBERs were expressed in the nucleus. B95–8 cells and Ts11 were used as positive and negative controls, respectively. Ts6 was an EBV-infection-negative tree shrew. No EBER-positive cells were detected inTs6 or Ts11.

### Immunohistochemical detection of EBV gene expression

**Fig. 9:**
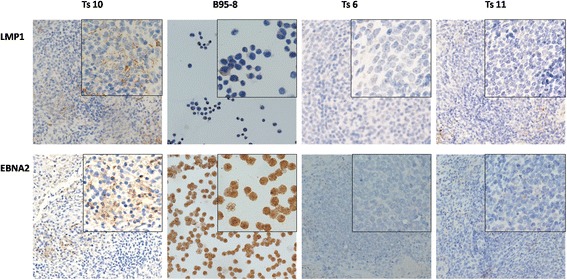
Immunohistochemical staining for the detection of EBV gene expression in spleens of tree shrews. LMP1 and EBNA2 expression were detected in Ts10.The positive cells were limited to the germinal centre.B95–8 cells and Ts11 were the positive and negative controls, respectively. Ts6 was an EBV-infection-negative tree shrew. No LMP1or EBNA2 expression was detected in Ts6 and Ts11

**Fig. 10:**
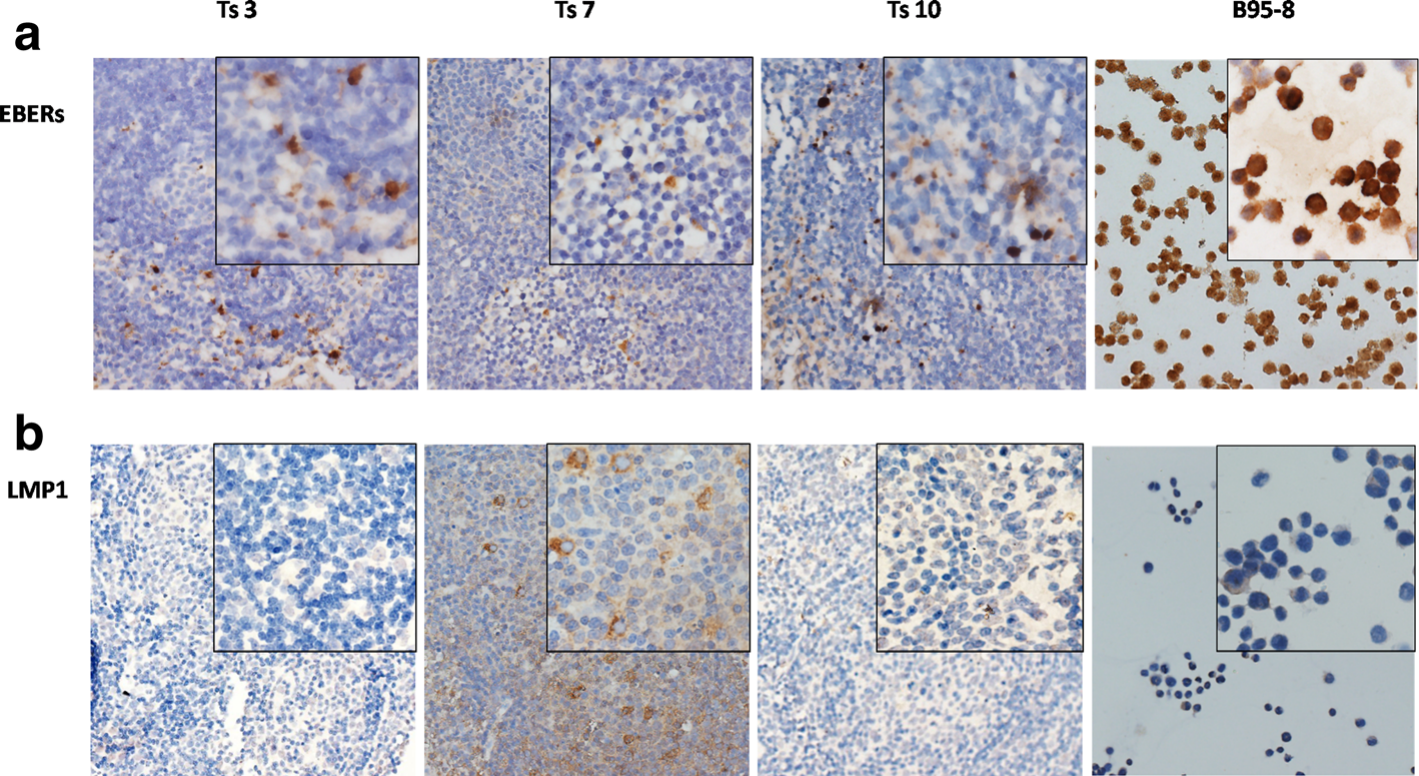
EBER-ISH and immunohistochemical staining of EBV gene expression in mesenteric lymph nodes of tree shrews. EBER-positive cells were detected in the mesenteric lymph nodes of three of the 10 tree shrews (**a**).LMP1 expression was detected in the mesenteric lymph nodes by immunohistochemistry only in Ts7 (**b**). No EBNA2 expression was detected in the mesenteric lymph nodes of the three tree shrews with mesenteric lymph node enlargement (data not shown)

The spleens, livers, mesenteric lymph nodes, lungs and nasopharyngeal tissues of the tree shrews were examined for LMP1 and EBNA2 expression using immunohistochemistry.LMP1- and EBNA2-positive cells were detected in the spleen of Ts10 only by immunohistochemistry. These positive cells were limited to the germinal centre (Fig. 9). In Ts7 only, LMP1 expression was detected in the mesenteric lymph nodes by immunohistochemistry (Fig. 10b). No EBNA2 expression was detected in the mesenteric lymph nodes of the three tree shrews with mesenteric lymph node enlargement (data not shown). LMP1 was expressed in the cytomembrane and cytoplasm, whereas EBNA2 was expressed in the nucleus. B95–8 cells and Ts11 were the positive and negative controls, respectively. Ts6 was an EBV-infection-negative tree shrew. No LMP1or EBNA2 expression was detected in Ts6 or Ts11.

### Western blotting for the detection of EBV gene expression

**Fig. 11:**
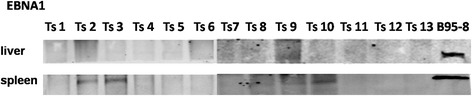
Western blotting for the detection of EBV gene expression in the livers and spleens. EBNA1 was detected in the spleens of Ts2, Ts3, Ts9 and Ts10. No EBNA1 expression was detected in the livers of any of the tree shrews. B95–8 cells and Ts11–13 were used as positive and negative controls, respectively. Ts5 and Ts6 were EBV-infection-negative. No EBNA1expression was detected inTs5, Ts6 or Ts11–13

We performed Western blotting to detect the expression of EBNA1 in the livers and spleens of the tree shrews. As shown in Fig. 11, EBNA1 expression was detected in the spleens of Ts2, Ts3, Ts9 and Ts10, but no EBNA1 expression was detected in the livers of any of the animals. B95–8 cells and Ts11–13 were used a spositive and negative controls, respectively. Ts5 and Ts6 were EBV-infection-negative. No EBNA1expression was detected inTs5, Ts6 or Ts11–13.

## Discussion

In this study, we inoculated10 tree shrews with EBV via intravenous infusion. Only eight tree shrews were successfully infected, albeit to different extents. The determination of EBV copy number in the PBMCs of tree shrews revealed that the infection observed in this study can be divided into three types: EBV infection-negative (Ts5 and 6);infection in which EBV copy number intermittently increased (Ts3,4 and 10); and infection in which EBV copy number transiently increased (Ts1,2,7,8 and 9). The differences in EBV copy number observed in the tree shrews in this study may be due to the different virus removal abilities of the tree shrews. In humans, infection with EBV stimulates CD23^+^B lymphocyte proliferation. The proliferated B cells replicate the virus [40] and stimulate CD4^+^T cell proliferation. During the stage of viral replication, CD4^+^T cells release lymphatic factors that modify the local inflammatory response and limit the proliferation of infected B cells, and CD4^+^T cells are greatly depleted [41]. Under conditions of intense viral antigen stimulation,CD8^+^T cells proliferate. The activity of CD8^+^T cells can also be greatly enhanced and can have a very strong cytotoxic effect.CD8^+^ T cells attack and destroy virus-infected CD23^+^B cells in body fluids and lymph nodes, inhibit their abnormal proliferation, release excessive cytokines and simultaneously cause symptoms of infectious mononucleosis (IM) [42], an EBV-associated disease. When the body’s immune system is functioning normally, most EBV copies are removed, and this may explain why the EBV copy numbers initially increased in some tree shrews but then decreased. However, EBV can occasionally escape the body’s immune surveillance through an unknown mechanism and attack the cells, resulting in a failure of the immune system to recognize and destroy virus-infected CD23^+^B cells. EBV can be present in latent form long-term in B lymphocytes or be reactivated when immune system function declines, resulting in a fluctuation in EBV copy number in the human body. Similar phenomena may have been responsible for the continuous increase in EBV copy number in some of the tree shrews in this study.

Using RT-PCR, we detected EBV-related gene expression after infection, including expression of the pyrolysis-related protein EBV BZLF1(Zta) and expression of the early gene (EA), both of which appear during the hyperplastic cracking stage of infection,as well as of related genes such as LMP1, EBNA1 and EBNA2 that appear in the latent infection stage. BZLF1 is a transcription factor of the cracking stage switch, and its expression occurs in the absence of the synthesis of any other viral proteins. BZLF1 may participate in the regulation of the cell cycle and cell proliferation [43], prompted by a change in viral infection from latent infection to lytic infection [19]. EA mainly appears during the acute phase of infection and is involved in viral DNA replication and blocking antigen presentation,among other roles [44]. Among the genes expressed after EBV expression, LMP1 is an important cancer-related gene and plays an important role in B cell and epithelial cell transformation, proliferation, and apoptosis [45]. In all 8 EBV-infection-positive tree shrews, BZLF1 and LMP1 were the easiest viral proteins to detect, and their expression was most typical in Ts3, Ts4, Ts7, and Ts10. The expression of EA and EBNA1 was detected occasionally in Ts7. In Ts7, for example, the EBV lytic protein gene BZLF1 was expressed at the beginning of infection, leading to viral replication, EA expression and an increase in the EBV copy number. However, later during infection, the body’s interventions, such as immune defences, result in a decrease in EBV copy number, expression absent of BZLF1. As the infection enters the latent phase, EBNA1 expression appears. This result is consistent with the histopathological results showing that EBER-positive and LMP1-positive cells were present in the spleen and the mesentericlymph nodes, findings that are compatible with the presence of latency II, III and lytic infection. Furthermore, different cells may have been in different latent phases. LMP1 was detected during the entire observation period in Ts7, suggesting that Ts7 was in a state of long-term latent infection. For Ts9,expression of EA and LMP1 appeared at week 2, a finding that is coincidental with the observation of maximal viral copy number inTs9 at week 2. Expression of EBNA1 appeared at week16 when no related genes had been expressed for several weeks, indicating that the infection had entered a latent stage resembling latency II infection. Although EBNA1 and EBNA2 are also important genes during EBV latent infection development [46], their expression was detected only occasionally in the 8 tree shrews. The most typical results were observed in Ts1 and Ts8. All gene detection was performed using B95–8 as a positive control and without template as a negative control.LMP was detected in the positive control and all samples as products of two sizes, 134 kb and/or 211 kb. By searching the NCBI gene bank, we confirmed that polypeptides of these two sizes can indeed be amplified by the same primers and that both are EBV-related gene products.

VCA IgG antibodies, which increase continuously from the acute phase of EBV infection in humans [47], were elevated to varying degrees after EBV vaccination in this experiment, similar to the previously reported changes in VCA IgG levels in the human body after EBV infection. Although EA IgG mainly appears during the acute infection period or during the reactivation phase [44], it was not elevated in any of the tree shrews in this study during an observation period of up to 21 weeks. EBNA IgG, which appears during the recovery period of EBV infection and persists at high levels [48], was not elevated in any of the tree shrews except Ts7. These observations suggest that the immune response of the tree shrew to EBV is different from that of humans. It is currently difficult to determine the exact reason for this difference. Therefore, it appears to be vitally important to determine the function of antibodies against VCA,EBNA and EA in tree shrews in vivo.

We did not detect VCA IgM, which in humans typically appears first, peaks during the acute infection period and is followed by a decline; this limitation, which was due to the lack of available anti-tree shrew IgM antibodies, should be addressed in future studies. Because secondary antibodies specific to tree shrews were not commercially available and the ELISA kits used in this study were developed for use with human serum, we commissioned Sangon Biotech company to synthesize a new secondary antibody and used it instead of the secondary antibody provided with the ELISA kits. The combination of a specific antibody with an ELISA kit has been applied in previous research [24]; the secondary antibody used in this study was shown to be specific and can be used in future research involving tree shrews.

We euthanized tree shrews after different observation periods ranging from 3 to 21 weeks; these time points may correspond to different stages of infection.Tree shrews that were infected by EBV for a long time were found to be in a state of latent EBV infection. The appearance of these tree shrews was normal, without obvious weakness; their condition may be similar to that of humans that have been shown to be long-term EBV carriers without any clinical manifestations. In the histopathological investigation, EBERs and LMP1- and EBNA2-positive cells were detected in the spleen. These cells were in the germinal centre, where viral and local tissue factors could mediate their effects and expansion [49].Consistent with a previous study showing that EBV infects B cells and epithelial cells, these cells appeared to be B lymphocytes; however,it is difficult to clarify whether the lymphocytes were B cells or T cells because of the absence of tree-shrew-specific reagents for the examination. EBER- and/or LMP1-positive cells were detected in the mesenteric lymph nodes of three tree shrews, and Western blotting detected EBNA1 in the spleens of Ts2, Ts3, Ts9 and Ts10. Furthermore, the detection of EBNA2 expression in the spleens of Ts10 but not in PBMCs or inliveror lung tissue again demonstrates that lymphoid tissue is the target site of EBV proliferation, consistent with the results described previously [50]. The EBV-related gene and protein expression suggest that tree shrews were in the latency I and II infection stages. However, the immune systems of the tree shrews without EBV-marker-positive cells in their tissues may have responded strongly to EBV and eliminated the virus from theanimals. Further characterization of the factors that help eliminate EBV could help in the treatment and prevention of EBV-related diseases. EBV can enter human epithelial cells via receptors [51, 52], through direct contact between cells [53, 54], by involvement of the virus envelope protein [55, 56], and through assistance mediated by cytokines [57]. However, in this study, we did not detect EBV-positive signals in the nasopharynges or lungs of the infected animals. The use of EBV intravenous inoculation may have prevented EBV from entering the lymphoid tissue,nasopharynx or oropharynx. Future studies should address whether EBV can enter the epithelial cells of tree shrews, whether the mechanism of EBV entry into the nasopharynx isthe same as that in humans, and the exact route of entry. The lung is not susceptible to EBV, whichmay explain why we failed to detect EBV-positive signals in the lung. These results further confirmed that the target cells of EBV are lymphocytes andthat the primary organ infected by EBV is the spleen.These results are consistent with those obtained in previousstudies.

## Conclusion

In this study, we preliminarily confirmed that EBV caninfect treeshrews after intravenous injection at a rate of 80%. This work establishes a foundation for the construction of an animal model of EBV infection in the future. Because of the similarity of tree shrews to primates, the tree shrew model of EBV offers some advantages for studying the infection and pathogenic mechanism of EBV and for the development of medicines and vaccines.

## Methods

### Animals

Thirteen adult (1–1.5 years of age) male and female tree shrews with body weights ranging from 120 to 160 g were obtained from the Experimental Animal Center of Kunming Medical University (Kunming, China). All animals were reared artificially since birth, housed under controlled environmental conditions with a 12 h light/dark photoperiod at the Laboratory Animal Center of Guangxi Medical University, and monitored by veterinarians. They were fed a complete formula food and provided water adlibitum. The experiment was begun after the tree shrews had undergone an adaptation period of at least one week.

### Preparation of EBV and inoculation of tree shrews

B95–8 cells (marmoset EBV-immortalized B-cell line) were cultured inRPMI1640 medium (Gibco, USA) supplemented with 10% foetal bovine serum (Gibco) and 1 × antibiotics (100 kU/l penicillin, 100 mg/l streptomycin, both from (Solarbio, Beijing, China)at 37 °C and 5% CO_2_in a humidified incubator. Growth medium was added to a sufficient volume when the cell density reached approximately 5 × 10^6^ cells/ml, and the cells were then cultured for 14 days. The cultures werefrozen at −80°Cand thawed 3 times in a water bath at 37 °C. Then, the supernatants were filtered using 0.45-μm filters to eliminate cells and centrifuged at 16,000 g at 4°Cfor 90 min to remove cell debris using a high-speed refrigerated centrifuge(Beckman Coulter,USA). After centrifugation, the pellets were resuspended in fresh RPMI 1640 medium and stored at −80 °C until tree shrew infection, as previously described.

### Inoculation of tree shrews with EBV

Ten tree shrews were inoculated with EBV by intravenous injection of 500μl of the viral solution described previously. The EBV copy number in the inoculums was approximately 3.9 × 10^8^ copies per animal as confirmed by real-time quantitative PCR(qPCR).The infectivity of the virus in the viral solution was assessed by its ability to successfully immortalize human PBMCs in vitro [25, 58]. The10 tree shrews inoculated with EBV were designated Ts1-Ts10. The week of viral inoculation was defined as “week 0.”As negative controls, three tree shrews (Ts11-Ts13) were inoculated with fresh RPMI 1640 medium containing 10% FBS using the same procedure.

### Collection of PBMCs and serum from the blood of tree shrews

Approximately 1.5 ml of blood was collected at regular intervals from the tree shrews in anticoagulative tubes containing EDTA•2Na(1 ml) and in sterile centrifuge tubes without anticoagulant(0.5 ml) from the femoral artery or vein. The anticoagulant blood was used to isolate peripheral blood mononuclear cells(PBMCs) using a Peripheral Blood Mononuclear Cell Separation Kit for Mice(Solarbio,Beijing, China); the blood collected in the other tubes was centrifuged at 3500 rpm at 4°Cin a centrifuge(Eppendorf,Germany) and the serum was carefully isolated. The serum samples were stored at −20 °C.

### Quantitative real-time PCR (qPCR) for the detection of EBV copy number

DNA was extracted from the initial inocula and from tree shrew PBMCs using the QIAamp DNA Mini Kit(Qiagen,Hilden, Germany)according to the manufacturer’s protocol. The EBV viral load in the original inocula and in the PBMCs was estimated using qPCR targeting the EBV BamHIW region. DNA from the Namalwa cell line (a Burkitt lymphoma cell line containing two copies of EBV), which was purchased from the Shanghai Institute for Biological Sciences, Chinese Academy of Sciences Institute of Cell Resource Center (Shanghai,China), was used as a standard as previously reported [59]. The culture conditions for the Namalwa cells and the DNA extraction methodology were as previously described. The quantity and purity of the extracted DNA were determined using a NanoDrop2000 instrument (Thermo Fisher Scientific,USA). Next, qPCR was performed on 2μl of template DNA in a total reaction volume of 20μl with *Premix ExTaq*™ (Probe qPCR) (Takara, Japan) using the described primers and a fluorogenic probe [59] synthesized by Takara Biotechnology Co. (Japan). All samples were analysed in duplicate in a 40-cycle reaction on an Applied Biosystems Step One Plus real-time PCR machine. All experiments were repeated 3 times independently, and the mean value obtained in the 3 experiments was considered the copy number of the samples.

### Reverse transcription polymerase chain reaction (RT-PCR) for the detection of EBV-related gene expression in PBMCs

Total RNA was extracted from tree shrew PBMCs with the RNAsimple Total RNA Kit (Tiangen, Beijing, China) according to the manufacturer’s protocol and quantified using a NanoDrop2000 instrument. Then, 500 ng of RNA was reverse transcribed to cDNA using the EasyScript One-Step gDNA Removal and cDNA Synthesis SuperMix (TransGen Biotech, Beijing, China).RT-PCR was performed with a PCR Mix(Invitrogen, USA) according to the manufacturer’s protocol. The expression of BZLF1,LMP1,EBNA1,EBNA2 and EA was determined by RT-PCR using the primers,including 4 primers we designed for RT-PCR, as follows:BZLF1: 5′-GCG GAC AAA AAT CAG GCG TT-3′ and 5′-GAA AAT GCC GGG CCA AGT TT-3′; LMP1:5′-AAT TTG CAC GGA CAG GCA TT-3′ and 5′-AAG GCC AAA AGC TGC CAG AT-3′; EBNA2: 5′-AAC TCC TGG CCC ATC CAA TG-3′ and 5′-GGA GGG GCG AGG TCT TTT AC-3′; EA: 5′-AAC GAG CAG ATG ATT GGG CA-3′ and 5′-CGT GGT GAC GTA GAG ATC CG-3′ and 1 primers previously reported [60]: EBNA1: 5′-GTC ATC ATC ATC CGG GTC TC-3′ and 5′-TTC GGG TTG GAA CCT CCT TG-3′.The “housekeeping gene” β-actin was amplified as an internal control using the primers previously described [61], and cDNA from B95–8 cells was used as a positive control.

### Enzyme-linked immunosorbent assay (ELISA) for the determination of EBV antibodies in tree shrew serum

EBV antibodies in tree shrew serum were measured using an enzyme-linked immunosorbent assay (ELISA).The anti-EBV capsid antigen(VCA) IgG,EBV nuclear antigen (EBNA) IgG, and early antigen (EA) IgG were measured with the Epstein-Barr virus VCA IgG ELISA Kit(Abnova,Taiwan, China),a human Epstein-Barr virus nuclear antigen (EBNA1) antibody (IgG) ELISA kit (CUSABIO, Wuhan, China), and a human Epstein-Barr virus early antigen (EBEA) antibody (IgG) ELISA kit (CUSABIO).Tree shrew serum diluted 1:10 with dilution buffer was placed into each plate, and bound antibodies were subsequently detected using rabbit anti-tree shrew IgGsecondary antibody synthesized by Sangon Biotech Co.,Ltd. (Shanghai, China). The antibody was diluted 1:3000 in5% defatted milk. After incubation at 37 °C for 45 min or 30 min,tetramethylbenzidine (TMB) solution or a mixture reagent of colour A and B was added, and colour was allowed to develop for 10 min in the dark.Then,the stop solution was added, and the absorbance of each well was measured at 450 nm.

### EBER-in situ hybridization(ISH) for the detection of EBV in tree shrew tissues

We detected EBER1 and EBER2 in tissues according to the EBER in situ hybridization protocol [62] and the EBER1、EBER2 probe sequences [63, 64] as previously described. Highly sensitive digoxigenin-labelled oligonucleotide probes were synthesized by Bioneer (Korea).Briefly, the process was performed on 5-μm sections of formalin-fixed,paraffin-embedded tissue sections of the spleens,livers,mesenteric lymph nodes,lungs, and nasopharynges of autopsied tree shrews. Sections of EBV-infected B95–8 cells were used as a positive control.Pre-hybridization was performed for 2-4 h to reduce nonspecific binding prior to hybridization overnight with a mixture of EBER-1 and EBER-2 probes. The hybridized probes were subsequently detected using the 2-step plus®Poly-HRP Anti-Mouse/Rabbit IgG Detection System from Zhongshan Goldenbridge Biotech (Beijing, China). Diaminobenzidinetetrahydrochloride (DAB) (ZhongshanGoldenbridge Biotech) was used as the chromogen. Other reagents, including proteaseK,preliminary hybridization solution,and oligonucleotide probe diluent, were provided by Boster Biotech(Wuhan, China).

### Pathological and immunohistochemical detection in tree shrews

Eleven of the 13 tree shrews were autopsied. Tissues, including the spleen, liver, mesenteric lymph nodes, lung and nasopharynx, were examined by haematoxylin and eosin (H&E) and immunohistochemistry for detecting the expression of EBV LMP1 and EBNA2 was performed on 5-μm paraffin-embedded tissue sections using mouse anti-LMP1 monoclonal antibody [CS 1–4] (Abcam, UK) and mouse anti-EBNA2 monoclonal antibody [PE2] (Abcam), both at 1/100 dilution, according to the protocol provided with the 2-step plus®Poly-HRP Anti- Mouse/Rabbit IgG Detection System and DAB detection system.

### Western blotting analysis of EBV gene expression in tree shrews

Proteins were extracted from spleen and liver tissues with 1× RIPA buffer (radioimmune precipitation buffer containing protease inhibitor cocktail set I, Beyotime, Shanghai, China), and the protein concentration was determined using an Enhanced BCA Protein Assay Kit (Beyotime). Western blotting analysis of EBV gene expression was performed using 80 μg of protein from the spleen and liver tissues of tree shrews, and GAPDH was used as an internal control. The primary antibodies used were mouse anti-EBNA1 monoclonal antibody (0211)(Thermo Fisher Scientific, USA) and mouse anti-GAPDH antibody at dilutions of 1/50 and 1/10000, respectively. The signals were detected using IRDye® 680RD goat-anti-mouse IgG (H + L) antibody (Licor, USA) at a dilution of 1/10000 and an Odyssey dual-colour infrared fluorescence imaging detection system (Licor).

## Abbreviations

ADV: Adenovirus
BRLF: BamHI R fragment leftward reading frame
BZLF: BamHI Z fragment leftward reading frame
EA: Early antigen
EBERs: EBV non-protein-encoding RNAs
EBNA: EBV nuclear antigen
EBV: Epstein–Barr virus
ELISA: Enzyme-linked immunosorbent assay
HAV: Hepatitis A virus
HBV: Hepatitis B virus
HCV: Hepatitis C virus
HE: Haematoxylin and eosin
HRV: Rotavirus
HSV: Herpes virus
huPBLs: Human peripheral blood lymphocytes
IHC: Immunohistochemistry
IM: Infectious mononucleosis
ISH: In situ hybridization
LCLs: Lymphoblastoid cell lines
LMP: Latent membrane protein;NK:Natural killer cell
NPC: Nasopharyngeal carcinoma
PBMCs: Peripheral blood mononuclear cells
PTLD: Post-transplant lymphoproliferative disorders
qPCR: Quantitative real-time PCR
RT-PCR: Reverse transcription polymerase chain reaction
SCID: Severe combined immunedeficiency
VCA: EBV capsid antigen
WB: Western blotting
